# MicroRNA biomarker identification for pediatric acute myeloid leukemia based on a novel bioinformatics model

**DOI:** 10.18632/oncotarget.4459

**Published:** 2015-07-01

**Authors:** Wenying Yan, Lihua Xu, Zhandong Sun, Yuxin Lin, Wenyu Zhang, Jiajia Chen, Shaoyan Hu, Bairong Shen

**Affiliations:** ^1^ Center for Systems Biology, Soochow University, Suzhou, 215006, China; ^2^ Taicang Center for Translational Bioinformatics, Taicang 215400, China; ^3^ The 100^th^ Hospital of PLA, Suzhou 215007, China; ^4^ Department of Hematology and Oncology, Children's Hospital of Soochow University, Suzhou 215003, China; ^5^ Department of Pediatrics, The First People's Hospital of Lianyungang, Lianyungang, Jiangsu 222002, China; ^6^ School of Chemistry, Biology and Material Engineering, Suzhou University of Science and Technology, Suzhou 215011, China

**Keywords:** microRNA biomarkers, transcription factor, pediatric acute myeloid leukemia, bioinformatics model, microRNA regulatory network

## Abstract

Acute myeloid leukemia (AML) in children is a complex and heterogeneous disease. The identification of reliable and stable molecular biomarkers for diagnosis, especially early diagnosis, remains a significant therapeutic challenge. Aberrant microRNA expression could be used for cancer diagnosis and treatment selection. Here, we describe a novel bioinformatics model for the prediction of microRNA biomarkers for the diagnosis of paediatric AML based on computational functional analysis of the microRNA regulatory network substructure. microRNA-196b, microRNA-155 and microRNA-25 were identified as putative diagnostic biomarkers for pediatric AML. Further systematic analysis confirmed the association of the predicted microRNAs with the leukemogenesis of AML. *In vitro* q-PCR experiments showed that microRNA-155 is significantly overexpressed in children with AML and microRNA-196b is significantly overexpressed in subgroups M4–M5 of the French-American-British classification system. These results suggest that microRNA-155 is a potential diagnostic biomarker for all subgroups of paediatric AML, whereas microRNA-196b is specific for subgroups M4–M5.

## INTRODUCTION

Acute myeloid leukemia (AML) is a rare and heterogeneous cancer that arises from the clonal transformation of hematopoietic precursors. It is the most common type of leukaemia diagnosed during infancy, accounting for 15–20% of cases of acute childhood leukemia. The overall survival (OS) rate of patients with AML has improved significantly in the last decades, and is currently in the range of 60–70% [1, 2]. Chemotherapy induces complete remission (CR) in approximately 90% of children. However, approximately one third of patients experience relapses with modern intensive chemotherapy protocols [2, 3]. Furthermore, the improvements in AML can be largely attributed to intensive use of conventional cytotoxic chemotherapy, whose late effects cause significant morbidity for many survivors. In contrast to the high OS rate (> 80%) of acute lymphocytic leukemia (ALL), the improvements in AML diagnosis and therapy have been limited. Therefore, novel approaches to the diagnosis and treatment of AML are needed [4].

MicroRNAs (miRNAs) are 18 to 22 nucleotide noncoding RNAs that regulate gene expression. They are predicted to silence over 60% of mammalian genes [5]. They are involved in a variety of critical biologic process, including cell cycle progression, differentiation, apoptosis, and immune responses [6–8]. miRNAs show aberrant expression patterns and functional abnormalities in cancers, and the expression pattern can be correlated with cancer type, stage, and other clinical variables [9]. Therefore, the identification of miRNAs may provide potential diagnostic biomarkers and therapeutic targets for cancer treatment.

In adult AML, several miRNAs display aberrant expression. Compared with healthy samples, miRNAs downregulated in AML could function as tumor suppressors, such as the miR-29 family. A comprehensive study investigated all members of the miR-29 family, including miR-29a, -29b and -29c. The downregulation of the miR-29 family during AML development results in the upregulation of their target proteins Akt2 and CCND2, which are involved in the regulation of cell proliferation and cell apoptosis [10]. miR-223 is downregulated in different subtypes of AML [11]. Downregulation of miR-193b upregulates the c-Kit proto-oncogene and represses cell proliferation in AML [12]. On the other hand, let-7e, miRNA-155 and miRNA-196b are overexpressed in adult AML patients [13–15]. However, there are important differences in both the diagnostic criteria and disease management between adults and children with AML [3]. The identification of miRNA biomarkers of paediatric AML remains limited. Furthermore, current efforts in biomarker identification have been directed at the detection of dysregulated miRNAs in miRNA expression profiles, which is not sufficient. Systematic methods that integrate information, such as microRNA regulatory data, gene expression profiles, and gene functional information are necessary to identify miRNA biomarkers.

POMA (Pipeline of Outlier MicroRNA Analysis), a prediction model previously developed by our group, could meet such needs [16]. This model defines a novel out degree (NOD), i.e., the number of genes exclusively targeted by certain microRNA (also known as unique target genes, UTGs) to measure the independent regulatory power of individual miRNAs [16–18]. Based on miRNA regulatory data and gene expression profiling data, POMA evaluates the relevance of miRNAs to given disease conditions, and it has been successfully applied to identify potential biomarkers in prostate cancer [18], clear cell renal cell carcinoma [16], and sepsis [17]. However, the POMA model does not consider the characteristics and function of the target genes of miRNAs, which play important roles in disease occurrence and development.

In the present study, we improved the POMA model by adding measurement of transcription factor percentage (TFP) of the microRNA target genes. There are three main reasons to include TFP measurement. First, the number of TFs targeted by a certain dysregulated miRNA is positively correlated with the number of downstream genes affected. Thus, dysregulated microRNAs with high regulatory potential of TFP may influence the expression of more genes directly or indirectly, and have a greater contribution to carcinogenesis. Second, TFs lie at the heart of various biological processes, such as DNA replication and repair, development, control of apoptosis, and cellular differentiation. Therefore, TF genes with aberrant expression will contribute to human carcinogenesis. Last but most important, most of the current studies about miRNA biomarker identification for cancer and other diseases are based on the global characters of the miRNA-mRNA network [19–21], and few studies have focused on the substructure of the network [22]. The substructures of the miRNA-mRNA network, such as miRNA-TF feed-forward loops and feedback loops, play an important role in cell proliferation, differentiation, and development, and are involved in several types of cancer [23].

**Figure 1:**
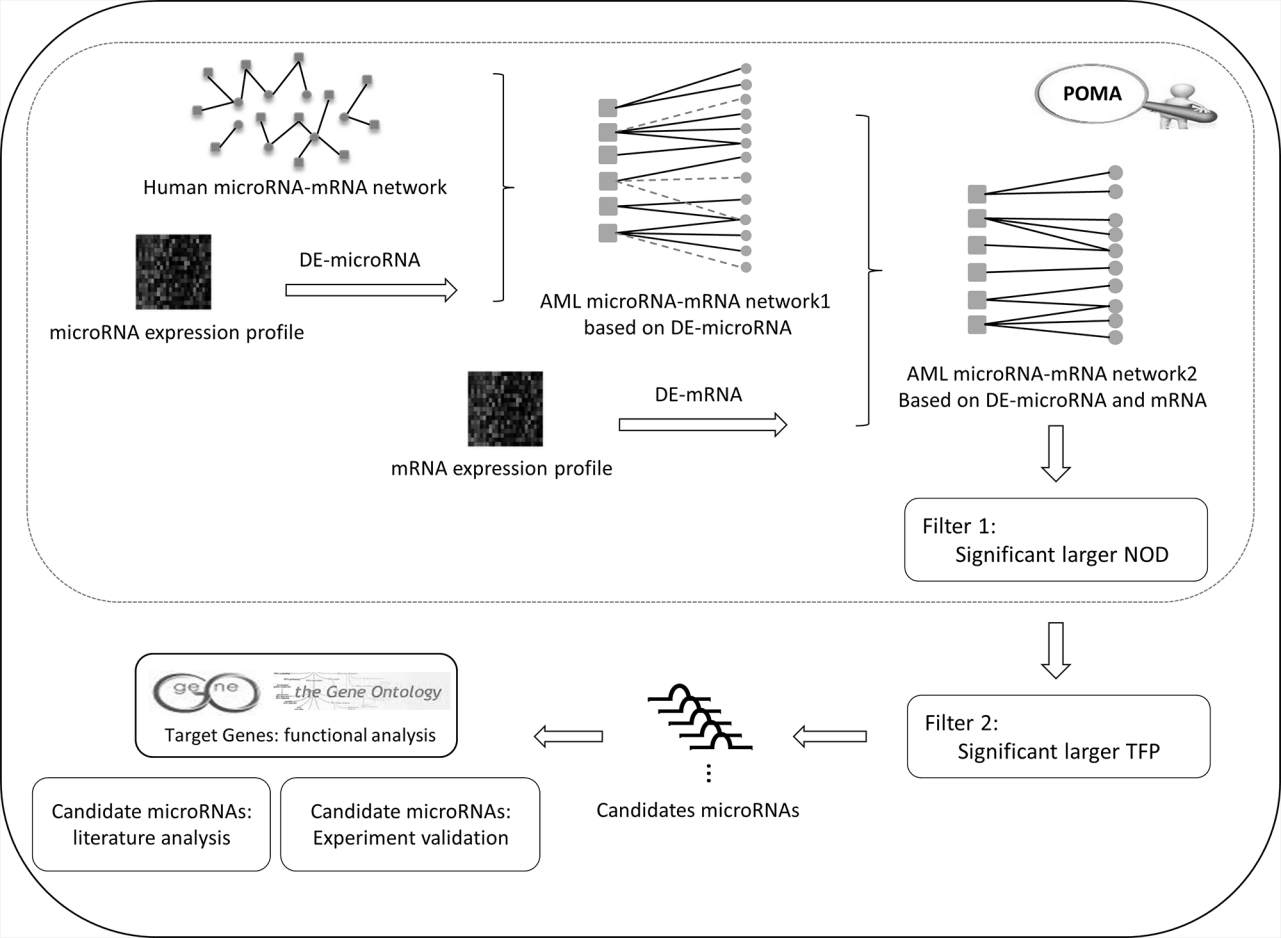
Analysis pipeline of the study

The improved POMA model is based on three hypotheses as follows: 1) miRNA activity could be reflected by the aberrant expression of its target genes; 2) miRNAs with larger NOD values are more likely to be biomarker candidates; and 3) in the miRNA set from hypothesis 2, miRNAs that target more transcription factors are more likely to be biomarkers. Using the model, we identified miR-196b, miR-155 and miR-25 as potential biomarkers for AML based on miRNA and mRNA expression profiles from paediatric patients. The analytical pipeline of this paper is shown in Figure 1.

## RESULTS

### Biomarkers display high NOD and Transcription Factor Percentage (TFP)

**Figure 2:**
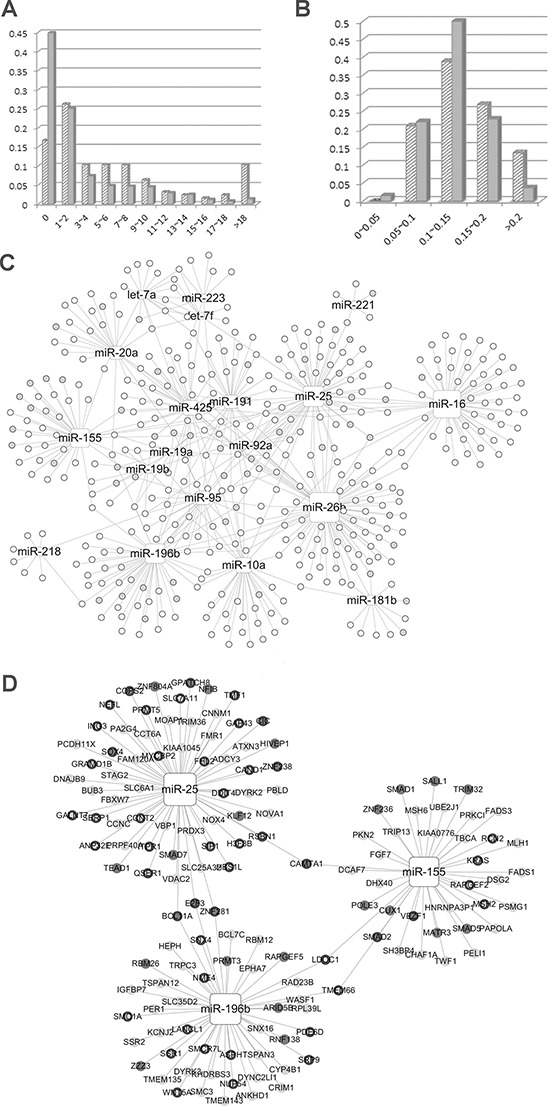
**A.** Novel out degree (NOD) distribution of microRNA (miRNA) biomarkers and other miRNAs. **B.** Transcription factor percentage (TFP) distribution of miRNA biomarkers and other miRNAs. In Figure 2A and 2B, biomarkers and others are represented by slashes and solid color, respectively. **C.** Paediatric acute myeloid leukaemia (AML) specific miRNA-mRNA network. **D.** candidate miRNAs with their target genes. Rectangle nodes represent miRNAs and the node size represents NOD values, i.e., the larger the node, the greater the NOD value. Circular nodes are genes and TF genes are marked by grey nodes. In Figure 2D, nodes without a black border are the genes exclusively regulated by a specific miRNA.

To confirm the NOD and TFP value distribution features of miRNAs, we investigated these two values for miRNAs in the context of the miRNA-mRNA network from the POMA model. A total of 126 miRNA biomarkers previously identified as biomarkers in 11 types of cancers were collected from the literature (Supplementary Table S4). As shown in our previous study, there is a statistically significant difference of NOD between potential biomarkers and other miRNAs (Wilcoxon test, *p* value = 3.416E-12) [18]. NOD distribution of the miRNA is shown in Figure 2A, which indicates that NOD values were positively correlated with the number of potential miRNA biomarkers. Therefore, we selected the miRNAs with the largest NOD values for future analysis and marked them as Set1.

To investigate the regulatory power of miRNA biomarkers, we defined a new parameter, namely TFP. The percentage of transcription factors among Set1 miRNA target genes was calculated and denoted as the TFP value. The biomarkers had significantly larger TFP values than the remaining miRNAs in Set1 (*p* = 0.0381, Wilcoxon test). TFP distribution is shown in Figure 2B, which indicates that the majority of miRNAs with larger TFP values (especially larger than 0.2) were potential biomarkers.

Our improved POMA method showed a better performance than the previous model. Using the previous POMA method, which identifies miRNA markers according to their NOD values, 203 miRNAs were identified as potential markers and 33% (67 miRNAs) had previously been reported as cancer biomarkers. According to the improved POMA model, which includes consideration of the NOD value and the TFP, 7 of the 10 identified miRNAs had previously been reported as biomarkers.

### Identifying candidate microRNA biomarkers for pediatric aml

Candidate miRNA biomarkers for pediatric AML were identified from the miRNA-mRNA association network. Firstly, the pediatric AML-specific miRNA-mRNA network (PAMLNet) was constructed from the expression profile (see methods) obtained with the POMA method. It comprised 531 links between 19 miRNAs and 406 genes. The network links are listed in Supplementary Table S5. The NOD and TFP values corresponding to each of the miRNAs in the PAMLNet were calculated and listed in Table 1. As shown in Figure 2C, the NOD values for all the miRNAs (rectangle nodes) were > 0 and the NOD values for certain miRNAs, such as miR-26b, miR-155, miR-196b, and miR-16 were large (the size of the square node represents the NOD value, i.e., the larger the node, the larger the NOD value). Some miRNAs had a greater number of associated TF genes (labeled as gray nodes), such as miR-155.

**Table 1:** miRNAs in the pediatric AML specific microRNA-mRNA network

miRNA	Targets Number	NOD	TFP (Number of TF)
let-7a	17	5	0.059 (1)
let-7f	12	2	0.083 (1)
microRNA-10a	35	20	0.057 (2)
microRNA-155	38	28	0.289 (11)
microRNA-16	59	48	0.068 (4)
microRNA-181b	12	8	0.250 (3)
microRNA-191	27	16	0.111 (3)
microRNA-196b	47	30	0.191 (9)
microRNA-19a	12	2	0.333 (4)
microRNA-19b	14	4	0.214 (3)
microRNA-20a	22	15	0.182 (4)
microRNA-218	8	6	0.000 (0)
microRNA-221	5	4	0.200 (1)
microRNA-223	8	4	0.125 (1)
microRNA-25	62	32	0.242 (15)
microRNA-26b	81	53	0.111 (9)
microRNA-425	32	19	0.125 (4)
microRNA-92a	11	1	0.182 (2)
microRNA-95	27	16	0.148 (4)

We select the miRNAs with significantly larger NOD values (Wilcoxon test, NOD > 23) and narrowed the list to a set of five miRNAs. Since only five miRNAs were detected, we selected those with the highest TFP values as the candidate biomarkers, and these were miR-196b, miR-155 and miR-25. The miRNAs analyzed and their corresponding target genes are shown in Figure 2D.

### Functional analysis of the target genes of candidate microRNA biomarkers

To explore the function of the predicted miRNA biomarkers and identify their regulated pathways in pediatric AML, we performed gene enrichment analysis using MetaCore from GeneGo (Thomson Reuters). The predicted candidate miRNA biomarkers, along with their regulated genes (see Supplementary Table S6), provided the potential miRNA-mRNA interaction pairs in AML.

**Figure 3:**
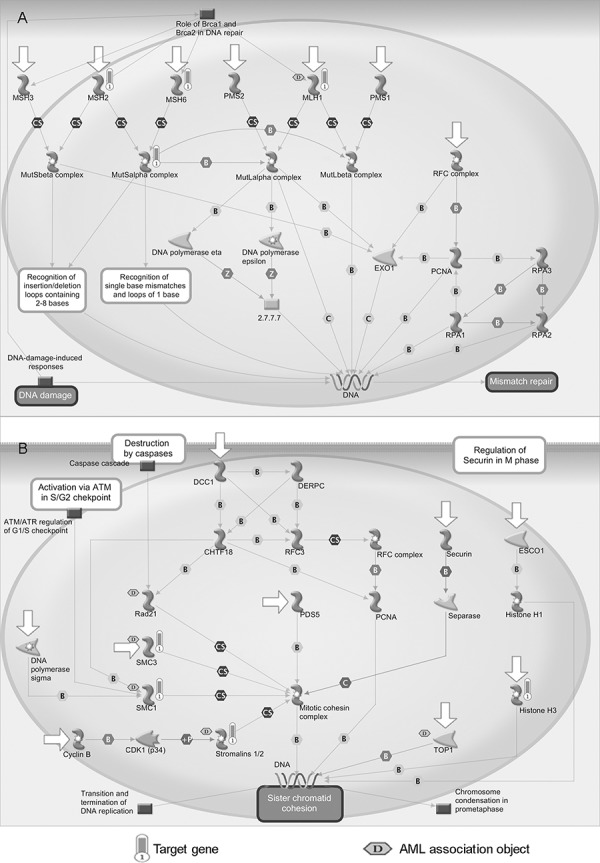
Enriched pathways of candidate miRNAs **A.** Mismatch repair pathway (belong to DNA damage). **B.** Sister chromatid cohesion pathway (belong to cell cycle). Objects with bars are mapped genes and those marked with a ‘D’ in the hexagon are associated with AML. Detail legends of the MetaCore pathways are provided in Supplementary Figure S1.

The enrichment analysis was performed on all target genes of miR-196b, miR-155 and miR-25. As shown in Figure 3A and 3B, The target genes were significantly enriched in two MetaCore pathways, namely mismatch repair (belonging to DNA damage) and sister chromatid cohesion (belonging to cell cycle) (*p* < 0.05 and FDR < 0.05). Four objects, namely MLH1, MSH2, MSH6 and the MutSalpha complex, were mapped in the mismatch repair pathway, of which MLH1 is associated with AML (see Figure 3A). Mismatch repair systems correct mismatches that form during DNA synthesis and genetic recombination, and as a result of DNA damage. Abnormalities of mismatch repair could play a key role in leukemogenesis, in particular in the development of AML [27]. Loss of the mismatch repair function is associated with refractory and relapsed AML and may contribute to disease pathogenesis [28].

Four objects were significantly enriched in the sister chromatid cohesion pathway, i.e., SMC3, SMC1, Stromalins 1/2 (STAG2), and histone H3 (see Figure 3B). Sister chromatid cohesion enables equal segregation of the duplicated genome to form daughter cells long after DNA replication has occurred [29]. Five objects were associated with AML, of which three were the mapped objects (SMC3, SMC1, and Stromalins 1/2) according to the MetaCore database. Recurrent mutations and deletions involving multiple components of the mitotic cohesion complex, including STAG2, RAD21, SMC1A and SMC3, were reported in different myeloid neoplasms. These mutations and deletions were mostly mutually exclusive and occurred in 12.1% of AML [30].

**Figure 4:**
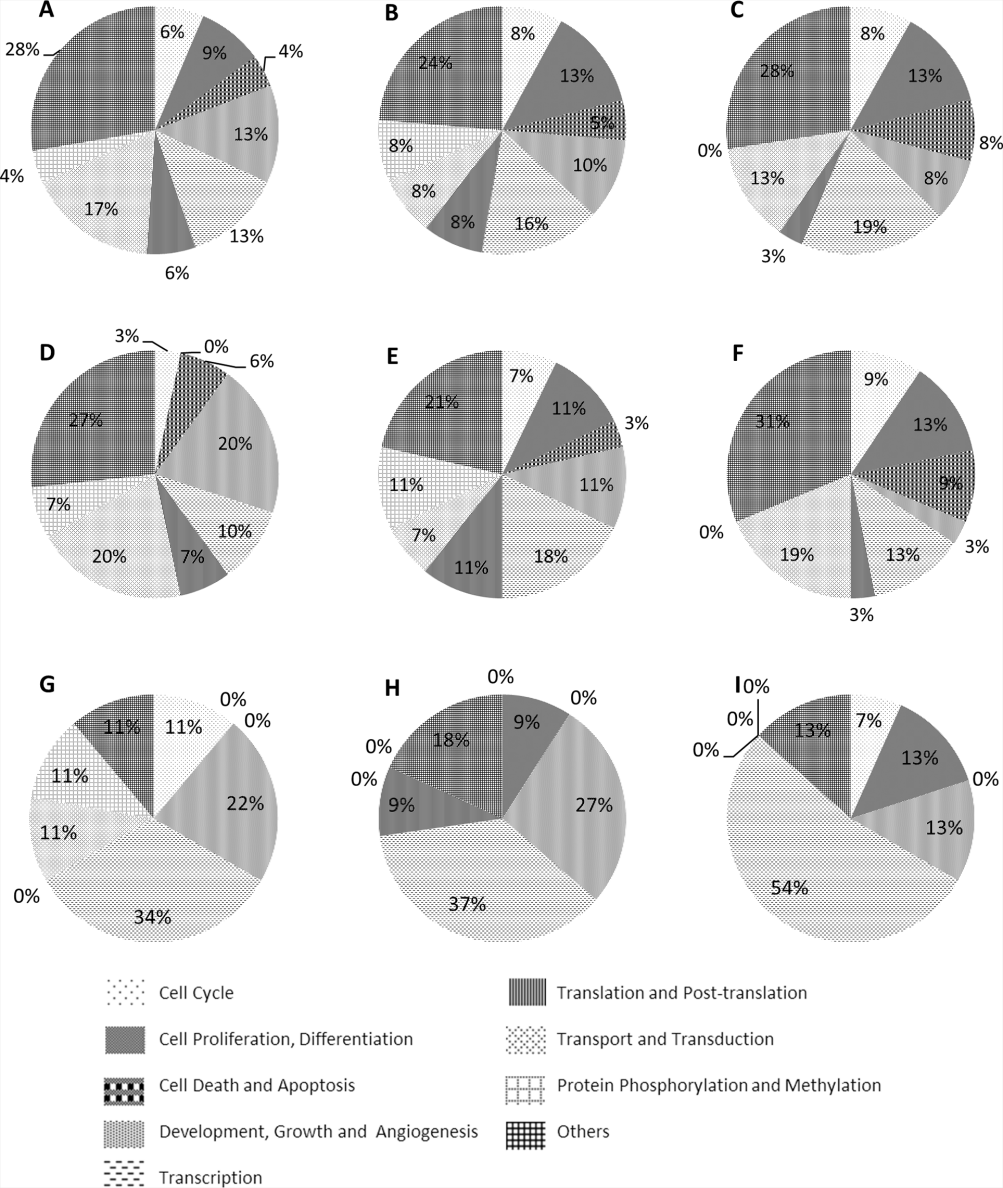
Pie charts of the biological processes of all target genes, unique target genes and TF genes of candidate miRNAs **A, B** and **C.** all target genes of miR-196b, miR-155 and miR-25. **D, E** and **F.** unique target genes of miR-196b, miR-155 and miR-25 **G, H** and **I.** TF genes of miR-196b, miR-155 and miR-25

Gene Ontology biological process classification of all target genes divided unique target genes (UTGs) and TF genes among the candidate miRNAs into nine groups, namely cell cycle, cell proliferation and differentiation, cell death and apoptosis, development, growth and angiogenesis, transcription, translation and post-translation, transport and transduction, protein phosphorylation and methylation and others (Figure 4). Most target gene groups for miR-196b, -155 and -25 (Figure 4A–4C) had a relatively even distribution among the corresponding unique and TF targets. However, a greater number of UTGs and TF genes of miR-196b (Figure 4D and 4G) and TF genes of miR-155 (Figure 4H) belonged to the development, growth and angiogenesis groups (>= 20%). More than one third of the TF genes of the three miRNAs were involved in the transcription group, and more than 30% of genes belonged to the cell cycle, proliferation and differentiation, cell death and apoptosis, and development, growth and angiogenesis groups, which are among the hallmarks of cancer [31] regardless of type. This indicates that miR-196b, miR-155 and miR-25 have a critical function in AML and may be potential biomarkers.

### *In vitro* and literature validation of candidate paediatric AML miRNA biomarkers

**Figure 5:**
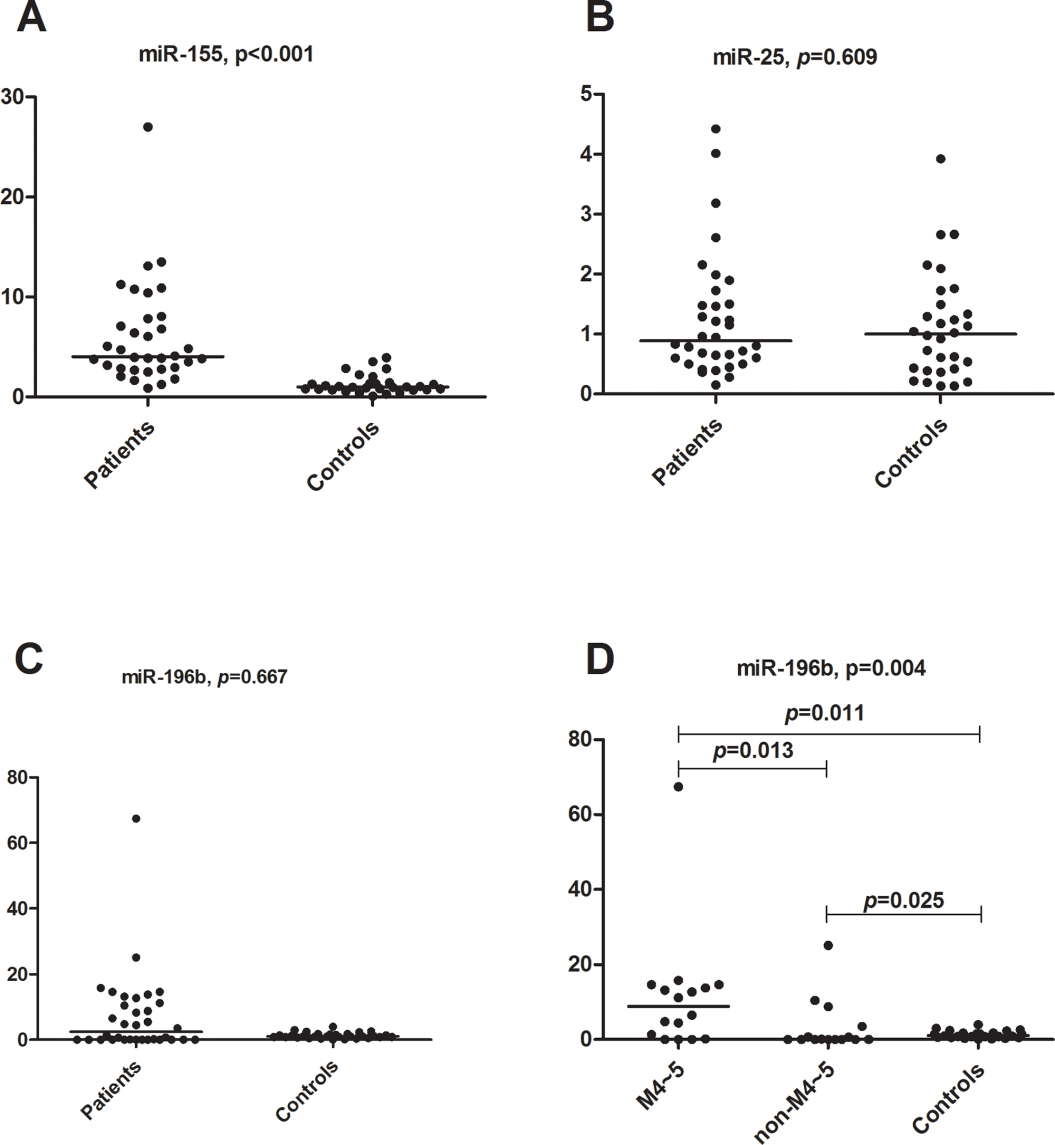
qRT-PCR results for miR-196b, miR-155 and miR-25 **A.** miR-155 relative expression comparison between primary childhood AML and non-malignant disease controls (Mann-Whitney *U* test, *p* < 0.001). **B.** miR-25 relative expression comparison between primary childhood AML and non-malignant disease controls (Mann-Whitney *U* test, *p* = 0.609). **C.** miR-196b relative expression comparison between primary childhood AML and non-malignant disease controls (Mann-Whitney *U* test, *p* = 0.667). **D.** miR-196b relative expression comparison between AML M4-M5 subtypes and controls (Mann-Whitney *U* test, *p* = 0.011), AML non M4-M5 subtypes and controls (*p* = 0.025), and AML M4-M5 subtypes and non-M4-M5 subtypes (*p* = 0.013), respectively. The comparison among these three groups was performed using the Kruskal-Wallis test (*p* = 0.004).

To further validate the three predicted miRNA biomarkers for paediatric AML, we assessed differences in their expression between pediatric AML and non-malignant disease samples using q-PCR technology. As shown in Figure 5, miR-155 was differentially expressed between the two groups of samples (*p* < 0.05), whereas no differential expression was detected for miR-196b and miR-25 (Figure 5A–5C). miR-155 was overexpressed in AML samples, and was therefore identified as a novel diagnostic biomarker for pediatric AML. Although miR-196b did not show significantly differences in expression between AML and controls, it was overexpressed in FAB M4-M5 samples when compared with controls (*p* = 0.011) and non-M4-M5 AML (*p* = 0.013) samples (Figure 5D). The expression of miR-196b in different samples is shown in Supplementary Table S7, and it suggests that miR-196b could be a diagnostic biomarker for AML subtypes M4-M5. Since miR-196b and miR-25 did not show significant outlier activity in paediatric AML, additional samples are needed to validate their roles.

We also performed a literature search of the three candidate miRNA markers to validate their relevance in the regulation of AML (Supplementary Table S8). Overexpression of miR-196b could result in increased proliferation, a partial differentiation block, and may contribute to leukemogenesis [32]. miR-196b is upregulated in adult AML patients compared to ALL patients and significantly associated with overall survival of AML patients [33]; therefore, it could serve as a prognostic marker for intermediate-risk cytogenetic AML [34].

miR-155 is a product of the B-cell integration cluster (BIC) and has significant impact on the biology of lymphocytes [35, 36]. It appears to play a role in myeloid differentiation [37] and immune function [38]. Sustained expression of miR-155 drives granulocyte/monocyte expansion in mice, and directly represses genes implicated in hematopoietic development [14]. It is upregulated in adult AML samples compared with healthy donors [14, 15].

miR-196b and miR-155 are associated with particular genetic subtypes, such as adult AMLs with FLT3 and NPM1 mutation [39–41], MLL rearrangements [42], as well as in pediatric AML patients [24].

## DISCUSSION

Since miRNAs can act both as oncogenes and tumor suppressor genes, their function is mostly regulatory and they are good candidates as cancer biomarkers [43, 44]. This underscores the need to develop methods to predict miRNA biomarkers. In our previous study, we defined an index termed NOD to measure the independent regulatory power of an individual miRNA [16–18]. In the present study, we introduced a new parameter, *i.e*. TFP, to measure the percentage of transcription factors regulated by a specific miRNA. Our results indicated that miRNAs with high NOD and TFP values were more likely to be biomarkers.

Based on this evidence, we improved the POMA method by taking into account the TF percentage among miRNA target genes to infer candidate biomarkers. The method was then applied to paediatric AML. Our results predicted miR-196b, miR-155 and miR-25 as biomarkers; we showed that miRNA-155 was overexpressed in AML samples and miR-196b was overexpressed in the M4-M5 subtype of AML *in vitro* by qRT-PCR. Although miR-196b and miR-25 did not show differential expression, miR-196b was previously reported to be aberrantly expressed in adult AML, especially in AML with FLT3 and NPM1 mutations.

There is limited information on miR-25, although it has been reported to be associated with overall survival of AML patients [33]. The involvement of miR-25 in human malignancies has been reported, including its role in promoting cell proliferation [45], regulating tumor cell apoptosis [46, 47], and promoting tumor invasion and metastasis [48]. It is aberrantly expressed in multiple cancers including gastric cancer [45], lung adenocarcinoma [49], and ovarian cancer [46] among others. Target genes of miR-25, such as STAG2, are abnormal in AML and may contribute to the development of AML [30, 50].

We performed a computational functional analysis of the target genes of three miRNAs. Two MetaCore pathways with significant enrichment of miRNA targets were found. An association between these pathways or their constituent objects with AML was previously reported. Moreover, more than one third of the targets of the predicted miRNAs are involved in biological processes that are among the hallmarks of cancer. The computational functional analysis supported our identification of miRNA biomarkers in pediatric AML.

The network-based approach of the present study identified potential biomarkers in pediatric AML and provides a systemic method to integrate different data. The co-regulation of miRNAs and TFs was not considered in the present model, and more detailed functional information of TFs needs to be integrated into our future models to improve the prediction.

Unbalanced datasets including more disease samples than healthy control samples may limit the power of the model for detecting true biomarkers. In the computation section of the present study, the published miRNA and mRNA datasets used contained fewer healthy controls than AML samples. To decrease the impact of this limitation, we included a higher number of control samples in the experimental validation section to make the number of AML and healthy control samples comparable, although the samples were difficult to harvest. In addition, the validation of conclusions depends on the quality of the input data. Our conclusions need to be validated in a large dataset in a future study. The objective of the present study was to identify putative miRNA biomarkers, and we used mRNA expression to infer the important miRNAs based on the miRNA-mRNA network. In the future, we could assess the protein expression levels of transcription factors to further optimize the model; however, at present, the protein level information is not required as input in our model.

In summary, we established a systematic level framework that integrates miRNA and mRNA expression data, and gene functional information based on POMA. The method identified miR-155 and miR-196b as promising potential diagnostic biomarkers for pediatric AML and AML subtype M4-M5, respectively.

## MATERIALS AND METHODS

### Dataset collection

The miRNA and mRNA expression datasets on pediatric AML were downloaded from the public database NCBI GEO. The miRNA expression dataset (GSE35320) comprises 102 pediatric AML samples, 6 adult AML samples and 2 healthy controls [24]. The present study used the 102 pediatric samples and 2 controls. Normalized miRNA data were downloaded directly for further analysis. In the mRNA expression dataset (GSE43176), there are 104 childhood AML samples and 4 healthy samples [25]. Detailed information of the two datasets is provided in Supplementary Table S1. The raw data of mRNA expression profiles were downloaded and then analysed using the ‘affy’ package from BioConductor with the RMA method. For genes with multiple probes, the average probe intensity was calculated.

### Prediction of pediatric AML microRNA biomarkers

Our improved POMA model was used to predict paediatric AML biomarkers. The previous version of POMA consists of four procedures as follows: detection of differential expressed miRNAs (DE-miRNA) and mRNAs (DE-mRNA), acquisition of inverse correlation pairs between miRNAs and mRNAs, construction of disease specific miRNA-mRNA networks and prioritizing disease-associated miRNA biomarkers [18]. In this study, the POMA model was improved as follows:

The DE-miRNAs and DE-mRNAs in AML samples and healthy controls were identified using the Limma R package [26]. As a result, 35 miRNAs and 3565 genes that were differentially expressed were identified (*p* < 0.05).

The aberrantly expressed miRNAs were mapped onto the human miRNA-mRNA network and labelled as the AML miRNA-mRNA network1. A human miRNA-mRNA network was constructed from experimentally validated data and computationally predicted data [16, 18]. Then, the differentially expressed genes showing a reverse expression pattern from miRNAs were mapped to the AML miRNA-mRNA network1 to generate the pediatric AML-specific miRNA-mRNA network. That is, AML microRNA-mRNA network2 in Figure 1.

Based on the pediatric AML specific miRNA-mRNA network, NOD values and TFP were used to measure the probability that a given miRNA would have a regulatory role in AML. NOD is the number of genes targeted exclusively by a specific miRNA. TFP is the percentage of transcription factors among the target genes of a miRNA. miRNAs with significantly larger NOD values than those of the other microRNAs were selected (Wilcoxon test, *p* < 0.05); among these, the miRNAs with significantly larger TFP values were selected as the potential miRNA biomarkers (Wilcoxon test, *p* < 0.05). In this study, five miRNAs with significantly larger NOD were identified, and the top three miRNAs according to the TFP values were selected.

### *In vitro* q-PCR confirmation of candidate AML microRNA biomarkers

The study included 34 children with AML and 30 with non-malignant disease aged 0–14 years. All samples were obtained from the bone marrow tissue bank of Children's Hospital of Soochow University. Detailed information on the samples is listed in Supplementary Tables S2 and S3. Mononuclear cells (MNCs) were isolated and stored at −80°C prior to RNA extraction. Informed consent was obtained from each participating individual's guardian. The study procedure was approved by the ethics committee of Children's Hospital of Soochow University.

Real-time quantitative PCR (TaqMan) was used to validate the expression of selected miRNAs biomarkers in individual bone marrow samples. miRNA (ABI, USA) levels were normalized using U6SnRNA as an internal control.

Total RNA was extracted using the Trizol reagent (Invitrogen, China). RNA was quantified on a MULTISKAN GO Microplate Spectrophotometer (Thermo Scientific, China). Universal reverse transcription for miRNAs was performed using the GeneAmp PCR System 9700 according to the manufacturer's instructions. The master mix for the cDNA synthesis contained 100 mM dNTP mix (0.15 μl), 50 U/μl MultiScribe RT enzyme (1 μl), 10 RT Buffer (1.5 μl), 20 U/μl RNase Inhibitor (0.19 μl), 5 TaqMan microRNA primers for each miRNA (3 μl) and nuclease free water up to 10 μl total volume; 10 ng of total RNA was used. qRT-PCRs for microRNA measurements were performed using the Taqman PCR Universal PCR Master Mix II no UNG (ABI) on a 7500 Real-Time PCR System (ABI). TaqMan miRNA assays (ABI) were used for detection of individual miRNAs using qRT-PCR according to the manufacturer's instructions (ABI). Quantitative PCR was performed in a volume of 20 μl containing 1.33 μl of cDNA, 10 μl of Taqman PCR Universal PCR Master Mix II no UNG, 1 μl of each primer, and 7.67 μl nuclease-free water. Triplicates were performed for all qRT PCR reactions. All quantitative PCR values were normalized to those of U6 snRNA. The relative expressions of three miRNAs were calculated using the 2^−ΔΔCt^ method. Statistical analyses were performed using SPSS 18.0 and Graphpad Prism software. The statistical significance of differences between two groups was calculated using the Mann-Whitney *U* test and that between three groups was calculated using the Kruskal-Wallis test.

### Functional analysis of target genes of candidate microRNA biomarkers

Enrichment analyses were performed using the MetaCore software from Thomson Reuters to examine the biological and functional relevance of miRNA target genes.

The threshold for significantly enriched pathways is *p* < 0.05 and FDR < 0.05. Each target gene was annotated by biological process in Gene Ontology.

## SUPPLEMENTARY DATA


