# Supplementary material for: MicroRNA biomarker identification for pediatric acute myeloid leukemia based on a novel bioinformatics model
Source: Oncotarget. 2015 Jul 1;6(28):26424–36. doi: 10.18632/oncotarget.4459 (PMC4694912; doi:10.18632/oncotarget.4459)
Supplement: Supplementary file 1 [file oncotarget-06-26424-s001.pdf]

SUPPLEMENTARY DATA

Supplementary Table S1. Summary of gene expression datasets used in this study

	MicroRNA Dataset	Gene Dataset
**GEO Accession**	GSE35320	GSE43176
**AML**	102	104
**Healthy**	2	4
**Age**	<18 years-old	Not available
**PMID**	23418555	23733505
**Tissue**	Blood Or Bone Marrow	Peripheral Blood: 31 Bone Marrow: 73

Supplementary Table S2. 34 primary childhood acute myeloid leukemia samples information

No	French-American-British classification	Sex	Month
AML 1	M5	Female	20
AML 2	M4	Female	95
AML 3	M4	Female	34
AML 4	M5	Male	124
AML 5	M5	Male	79
AML 6	M4	Female	129
AML 7	M6	Male	120
AML 8	M2	Female	43
AML 9	AML	Female	88
AML 10	M5	Female	15
AML 11	M5	Female	24
AML 12	M5	Female	31
AML 13	M4	Female	12
AML 14	M2	Male	157
AML 15	M5	Female	35
AML 16	M2	Female	117
AML 17	M3	Male	121
AML 18	M5	Female	67
AML 19	M4	Female	84
AML 20	M4	Female	112
AML 21	M2	Male	73
AML 22	M2	Male	87
AML 23	M2	Female	100
AML 24	M5	Male	71
AML 25	AML	Female	17
AML 26	M2	Male	98
AML 27	M2	Female	60
AML 28	M5	Male	6
AML 29	M2	Male	109
AML 30	M2	Male	116
AML 31	AML	Male	4
AML 32	M2	Male	91
AML 33	M2	Male	146
AML 34	M2	Male	118

Supplementary Table S3. 30 control samples information

No	non-malignant Disease	Sex	Month
1	infectious disease	M	60
2	infectious disease	M	12
3	ITP	F	84
4	ITP	F	84
5	ITP	F	108
6	ITP	M	24
7	ITP	M	72
8	infectious disease	F	6
9	ITP	F	18
10	ITP	F	120
11	ITP	M	1
12	ITP	F	12
13	ITP	M	60
14	ITP	F	60
15	ITP	F	84
16	ITP	F	72
17	ITP	M	72
18	ITP	F	24
19	infectious disease	F	120
20	ITP	F	132
21	ITP	M	36
22	infectious disease	F	12
23	ITP	F	12
24	infectious disease	M	72
25	ITP	M	48
26	ITP	M	120
27	hemolytic anemia	M	12
28	ITP	M	84
29	healthy donor	M	72
30	ITP	F	84

ITP: idiopathic thrombocytopenic purpura

Supplementary Table S4. Previously reported microRNA biomarkers in Cancer

ID	Cancer Name	Sample Type	Origin	PMID	Year	Expression Pattern	Validation Method
let-7b	Pca	Tissue	Germany	23798998	2013	Down	qRT-PCR
let-7d	Pca	Tissue	China	20873592	2010	Down	ISH and TMA
let-7f	LC	Serum	China	24697119	2014	Down	Real-time qRT-PCR
let-7g	Pca	Tissue	China	20873592	2010	Down	ISH and TMA
miR-1	GC	Serum	China	21112772	2011	Up	qRT-PCR
miR-1	Pca	Cell lines Tissue	USA	22210864	2012	Down	qRT-PCR
miR-1	LC	Serum	Germany	23810247	2013	Down	Real-time qRT-PCR
miR-100	Pca	Tissue	Brazil	21255804	2011	Up	qRT-PCR
miR-100	Leukaemia	Bone marrow mononuclear cells	China	23055746	2012	Up	Real-time qRT-PCR
miR-100	LC	Tissue	China	23842624	2013	Down	qRT-PCR
miR-100	EC	Tissue	China	24651474	2014	Up	qRT-PCR
miR-101	CRC	Tissue	USA	22930392	2013	Down	RT-PCR
miR-101	LC	Tissue	China	23178713	2012	Down	qRT-PCR
miR-101	LC	Serum Tissues	China	24260081	2013	Up	Real-time qRT-PCR
miR-105	Pca	Cell lines	Canada	23950948	2013	Up	Real-time qPCR
miR-106a	GC	Tissue	China	18996365	2009	Up	Real-time qRT-PCR
miR-106a	GC	Tissue	China	23335180	2013	Up	Real-time qRT-PCR
miR-106a	CRC	Fecal occult blood	Japan	23950216	2013	Up	Real-time qRT-PCR
miR-106b	GC	Plasma	China	23307259	2013	Up	qRT-PCR
miR-106b	GC	Serum	Japan	24104965	2013	Up	qRT-PCR
miR-107	GC	Tissue	Japan	22407237	2012	Up	Real-time PCR-based miRNA profiling array
miR-10b	PC	Tissue	USA	21652542	2011	Up	Fluorescence-based ISH
miR-10b	CRC	Tissue	Japan	22322955	2012	Up	Real-time qRT-PCR
miR-122	LC	Serum	China	22174818	2011	Up	qRT-PCR
miR-122	CRC	Tissue	Japan	23373973	2013	Up	RT-PCR
miR-122	LC	Serum	Germany	23810247	2013	Down	Real-time qRT-PCR
miR-122	GC	Plasma	China	24481716	2014	Down	qRT-PCR
miR-122a	LC	Serum	China	23723713	2013	Down	qRT-PCR
miR-124	LC	Cell lines	China	24211205	2013	Up	Real-time qPCR

(*Continued*)

ID	Cancer Name	Sample Type	Origin	PMID	Year	Expression Pattern	Validation Method
miR-1246	EC	Serum	Japan	23361059	2013	Up	Real-time qPCR
miR-1247	PC	Tissue	China	24588767	2014	Down	ISH
miR-125a	LC	Tissue	China	22768249	2012	Up	Real-time qPCR
miR-125a-3p	GC	Tissue	Japan	22322911	2012	Down	Real-time qRT-PCR
miR-125a-5p	GC	Tissue	Japan	21220473	2011	Down	Real-time qRT-PCR
miR-125b	CRC	Tissue	Japan	21399871	2011	Up	Real-time qRT-PCR
miR-125b-5p	LC	Plasma	Turkey	24595450	2014	Up	qRT-PCR
miR-126	Leukaemia	Peripheral blood mononuclear cells	Japan	22884882	2012	Down	qRT-PCR
miR-126	Pca	Tissue	China	24350576	2013	Down	qRT-PCR
miR-126	CRC	Tissue	China	24532280	2014	Down	Real-time qPCR
miR-126	CRC	Serum	China	24653631	2014	Down	Real-time qPCR
miR-130b	LC	Serum	Hong Kong	22403344	2012	Up	Real-time qRT-PCR
miR-130b	PC	Tissue Cell line	China	24040078	2013	Down	Real-time qRT-PCR
miR-130b	CRC	Cell lines	USA	24204200	2013	Up	qRT-PCR
miR-133b	CRC	Tissue	Sweden	21573504	2011	Down	Real-time qPCR
MiR-133b	Pca	Tissue	China	24610824	2014	Up	Real-time qRT-PCR
miR-135a	Leukaemia	Bone marrow Peripheral blood	Spain	24072101	2013	Down	Real-time qPCR
miR-137	CRC	Tissue	China	23275153	2013	Down	Real-time qRT-PCR
miR-138	NC	Tissue	China	22739938	2012	Down	Real-time qRT-PCR
miR-139	LC	Plasma	China	24549282	2014	Down	qRT-PCR
miR-141	CRC	Plasma	USA	21445232	2011	Up	qRT-PCR
miR-141	Pca	Plasma	USA	21723797	2011	Up	qRT-PCR
miR-141	Pca	Serum	USA	22887127	2013	Up	Real-time qPCR
miR-141	CRC	Serum	China	24653631	2014	Up	Real-time qPCR
miR-142-3p	Leukaemia	Peripheral blood mononuclear cells	China	21678057	2012	Down	Real-time qRT-PCR

(*Continued*)

ID	Cancer Name	Sample Type	Origin	PMID	Year	Expression Pattern	Validation Method
miR-142-5p	GC	Tissue	China	21343377	2011	Down	Real-time qPCR
miR-143	Pca	Tissue	China	21647377	2011	Down	qRT-PCR
miR-143	GC	Tissue	China	21874264	2011	Down	Real-time qRT-PCR
miR-143	CRC	Tissue	China	22273643	2012	Down	TaqMan miRNA assays
miR-143	PC	Tissue	Italy	22836856	2012	Down	Real-time qRT-PCR
miR-143	GC	Tissue	Japan	24283360	2014	Up	qRT-PCR
miR-144	CRC	Tissue	Japan	22983984	2012	Down	Real-time qRT-PCR
miR-144*	CRC	Tissue	Italy	21863218	2011	Up	Real-time qRT-PCR
miR-145	Pca	Tissue	China	21647377	2011	Down	qRT-PCR
miR-145	CRC	Tissue	China	22273643	2012	Down	TaqMan miRNA assays
miR-145	Pca	Tissue	Greece	23703249	2013	Down	Real-time qPCR
miR-146a	GC	Tissue	China	22020746	2012	Up	qRT-PCR
miR-146a	LC	Tissue	China	24172202	2014	Down	Real-time qRT-PCR
miR-146b-3p	Pca	Tissue	Australia	23846169	2013	Up	qRT-PCR
miR-148a	LC	Tissue	Hong Kong	22496917	2012	Up	Real-time qRT-PCR
miR-148a	CRC	Tissue	Spain	23056401	2013	Down	Real-time qRT-PCR
miR-148a	PC	Tissue	USA	23933230	2013	Down	Real-time qRT-PCR
miR-148a	GC	Tissue	China	24527072	2014	Down	qPCR
miR-150	CRC	Tissue	China	22052060	2012	Down	qRT-PCR
miR-150	Leukaemia	Plasma	Belgium	23391324	2013	Down	real-time qRT-PCR
miR-155	PC	Pancreatic juicetissue	Japan	21068491	2010	Up	Real-time qRT-PCR
miR-155	CRC	Tissue	Japan	21412018	2010	Up	RT-qPCR
miR-155	LC	Tissue	China	22071603	2012	Up	qRT-PCR
miR-155	LC	Tissue	Taiwan	22629365	2012	Up	Real-time qRT-PCR
miR-155	PC	Tissue	Greece	22850622	2013	Up	Real-time qRT-PCR
miR-155	Leukaemia	Peripheral blood mononuclear cells	Japan	22884882	2012	Up	qRT-PCR
miR-155	LAC	Tissue	China	23437123	2013	Up	Real-time qPCR
miR-155	GEDC	Tissue	Japan	23660842	2013	Up	real-time qRT-PCR
miR-155	LC	Tissue	China	23863669	2013	Up	qPCR

(*Continued*)

ID	Cancer Name	Sample Type	Origin	PMID	Year	Expression Pattern	Validation Method
miR-15b	LC	Serum	Hong Kong	22403344	2012	Up	Real-time qRT-PCR
miR-16	Leukaemia	Lymph node	China	23260326	2013	Up	Real-time qRT-PCR
miR-16	CRC	Tissue	China	24045965	2013	Down	qRT-PCR
miR-16	GC	Plasma	China	24595006	2014	Up	qRT-PCR
miR-16	LC	Serum	China	24697119	2014	Down	Real-time qRT-PCR
miR-17	CRC	Tissue	China	22065543	2012	Up	Real-time qRT-PCR
miR-17	NC	Serum	China	23056289	2012	Up	Real-time qPCR
miR-17-5p	GC	Plasma	China	22406928	2012	Up	Real-time qRT-PCR
miR-17-5p	LC	Tissue	China	22583011	2012	Up	qRT-PCR
miR-17-5p	LC	Serum	China	23108086	2013	Up	qRT-PCR
miR-181b	Leukaemia	Leukemic cells	USA	21636858	2011	Down	Real-time qPCR
miR-181b	PC	Stool	China	22504911	2012	Up	qRT-PCR
miR-181b	GC	Tissue	China	21876743	2011	Up	Real-time qRT-PCR
miR-181b-5	Leukaemia	Serum	China	23437222	2013	Up	qRT-PCR
miR-181c	GC	Tissue	China	23425811	2013	Up	real-time qRT-PCR
miR-181c	GC	Plasma	China	23803080	2013	Up	Real-time qRT-PCR
miR-182	Pca	Tissue	China	20873592	2010	Down	ISH and TMA
miR-182	GC	Tissue	China	21874264	2011	Up	Real-time qRT-PCR
miR-182	LC	Tissue	China	22681717	2012	Up	qRT-PCR
miR-182	CRC	Tissue	China	23474644	2013	Up	Real-time qPCR
miR-182-5p	Pca	Tissue	Japan	23184537	2013	Up	Real-time qRT-PCR
miR-182-5p	Pca	Tissue	USA	23383207	2013	Up	real-time qRT-PCR
miR-183	Pca	Tissue	China	20873592	2010	Down	ISH and TMA
miR-183	Pca	Cell lines	USA	23538390	2013	Up	Real-time qPCR
miR-183	CRC	Tissue	China	24150523	2014	Up	Real-time qPCR
miR-184	TSCC	Tissue	Hong Kong	18451220	2008	Up	Real-time qPCR
miR-185	CRC	Tissue	Sweden	21573504	2011	Up	Real-time qPCR
miR-185	LC	Tissue	China	23648054	2013	Up	qRT-PCR
miR-18a	LC	Serum	China	22865399	2012	Up	Real-time qRT-PCR
miR-18a	NC	Cell linesTissue	China	23097559	2013	Up	Real-time qRT-PCR
miR-18a	EC	Plasma	Japan	23579215	2013	Up	qRT-PCR
miR-18a	CRC	Tissue	Spain	23673725	2013	Up	Real-time qPCR

(*Continued*)

ID	Cancer Name	Sample Type	Origin	PMID	Year	Expression Pattern	Validation Method
miR-18a	GC	Plasma	Japan	24626859	2014	Up	qRT-PCR
miR-18a	CRC	Plasma	China	24649179	2013	Up	Real-time qRT-PCR
miR-18b	LC	Tissue	Japan	23496901	2013	Up	Real-time qPCR
miR-191	GC	Tissue Serum	China	24603541	2014	Up	qRT-PCR
miR-192	GC	Tissue	China	22205577	2012	Down	Real-Time PCR
miR-192	PC	Tissue	China	23612862	2013	Up	Real-time qPCR
miR-192	GC	Plasma	China	24481716	2014	Up	qRT-PCR
miR-192/ miR-194	PC	Tissue	China	24398877	2014	Up	Real-time qPCR
miR-194	Pca	Tissue	Australia	23846169	2013	Up	qRT-PCR
miR-195	CRC	Tissue	China	21390519	2012	Up	Real-time qRT-PCR
miR-195	GC	Tissue	China	21987613	2011	Up	Real-time qRT-PCR
miR-195	GC	Tissue	Israel	22046085	2011	Up	Real-Time qPCR
miR-196a	EC	Tissue	USA	19342367	2009	Up	Real-time qPCR
miR-196a	PC	Serum	China	20614181	2011	Up	Real-time qRT-PCR
miR-196a	PC	Tissue	USA	23933230	2013	Up	Real-time qRT-PCR
miR-196a	LAC	Tissue	Japan	23967217	2013	Up	Real-time qPCR
miR-196a	GC	Tissue	China	24527072	2014	Up	qPCR
miR-196b	PC	Tissue	USA	22114139	2012	Up	Real-time qRT-PCR
miR-196b	Leukaemia	Bone marrow; Peripheral blood	Spain	24072101	2013	Up	Real-time qPCR
miR-198	PC	Tissue	USA	23989979	2013	Down	Real-time qRT-PCR
miR-199a-3p	GC	Tissue	Israel	22046085	2011	Up	Real-Time qPCR
miR-199a-3p	GC	Plasma	China	22956063	2013	Up	Real-time qRT-PCR
miR-199a-3p	CRC	Tissue	China	23292866	2013	Up	Real-time qRT-PCR
miR-199a-3p	GC	Tissue	China	23733518	2013	Up	Real-time qRT-PCR
miR-19a	LAC	Tissue	China	24427326	2013	Up	qPCR
miR-20	Pca	Tissue	China	22994753	2012	-	-
miR-200b	Pca	Tissue	China	24317363	2014	Down	Real-time qRT-PCR
miR-200c	CRC	Tissue	Germany	18079988	2006	Up	qRT-PCR
miR-200c	PC	Serum	Japan	20579395	2010	Up	Real-time qRT-PCR

(*Continued*)

ID	Cancer Name	Sample Type	Origin	PMID	Year	Expression Pattern	Validation Method
miR-200c	CRC	Tissue	Japan	22735571	2013	Up	Real-time qPCR
miR-200c	GC	Plasma	Spain	22954417	2012	Up	Real-time qRT-PCR
miR-200c	CRC	Tissue	Japan	23982750	2014	Up	qRT-PCR
miR-200c	CRC	Plasma	China	24649179	2013	Up	Real-time qRT-PCR
miR-203	PC	Tissue	Japan	20652642	2010	Up	qRT-PCR
miR-203	Pca	Cell lines	Italy	21368580	2011	Down	qRT-PCR
miR-203	LC	Tissue	China	21786180	2012	Down	qRT-PCR
miR-203	EC	Tissue	NA	24519530	2014	Down	NA
miR-203	PC	Cell line	China	24520289	2014	Up	qPCR
miR-203	LAC	Tissue	China	24682952	2014	Down	qRT-PCR
miR-205	EC	Cell lines	Japan	20428818	2010	Up	Real-time qRT-PCR
miR-205	Pca	Tissue	Australia	22869146	2013	Down	qRT–PCR
miR-205	Pca	Tissue	USA	24167554	2013	Down	Real-time qPCR
miR-205	LAC	Tissue	China	24297308	2014	Down	Real-time qPCR
miR-206	GC	Tissue	China	23751352	2013	Down	qRT-PCR
miR-20a	GC	Serum	China	21112772	2011	Up	qRT-PCR
miR-20a	Pca	Plasma	USA	22298119	2012	Up	qRT-PCR
miR-20a	GC	Plasma	China	22406928	2012	Up	Real-time qRT-PCR
miR-20a	NC	Serum	China	23056289	2012	Up	Real-time qPCR
miR-20a	GC	Plasma	China	23307259	2013	Up	qRT-PCR
miR-20a	LC	Tissue	China	23594563	2013	Down	Real-time qPCR
miR-21	GC	Tissue	China	18507035	2008	Up	qPCR
miR-21	TSCC	Tissue	China	19509158	2009	Up	qRT-PCR
miR-21	CRC	Tissue	USA and HK	19737943	2009	Up	qRT-PCR
miR-21	EC	Tissue	USA and Japan	19789312	2009	Up	qRT-PCR
miR-21	PC	Pancreatic juice Tissue	Japan	21068491	2010	Up	Real-time qRT-PCR
miR-21	CRC	Tissue	Japan	21412018	2010	Up	RT-qPCR
miR-21	LC	Plasma	Japan	21749846	2012	Up	qRT-PCR
miR-21	GC	Tissue	China	21876743	2011	Up	Real-time qRT-PCR
miR-21	CRC	Stool samples	China	21930727	2014	Up	Real-time qRT-PCR
miR-21	Pca	Tissue	China	22341810	2012	Up	ISH and TMA

(*Continued*)

ID	Cancer Name	Sample Type	Origin	PMID	Year	Expression Pattern	Validation Method
miR-21	GC	Serum	China	22430134	2011	Up	Real-time qRT-PCR
miR-21	PC	Tissue	Italy	22836856	2012	Down	Real-time qRT-PCR
miR-21	PC	Tissue	Greece	22850622	2013	Up	Real-time qRT-PCR
miR-21	GC	Plasma	China	22860003	2012	Up	qRT-PCR
miR-21	CRC	Plasma	USA	22868372	2012	Up	Real-time qPCR
miR-21	PC	Tissue	Italy	23139258	2013	Up	qRT-PCR
miR-21	LAC	Tissue	China	23259291	2012	Up	qRT-PCR
miR-21	GC	Plasma	Japan	23267156	2013	Up	qRT-PCR
miR-21	GC	Tissue	China	23335180	2013	Up	real-time qRT-PCR
miR-21	CRC	Serum	China	23625654	2013	Up	real-time qRT-PCR
miR-21	CRC	Serum	Japan	23704278	2013	Up	qRT-PCR
miR-21	CRC	Serum	Spain	23970420	2013	Down	qRT-PCR
miR-21	GC	Serum	Japan	24104965	2013	Up	qRT-PCR
miR-21	GEDC	Plasma	Japan	24118467	2013	Up	qRT-PCR
miR-21	CRC	Tissue	Japan	24122631	2014	Up	qRT-PCR
miR-21	GC	Plasma	China	24460332	2013	Down	Real-time qRT-PCR
miR-21	EC	Tissue	NA	24519530	2014	Up	NA
miR-21	CRC	Serum	China	24653631	2014	Up	Real-time qPCR
miR-21	LC	Serum	China	24697119	2014	Down	Real-time qRT-PCR
miR-210	PC	Plasma	USA	20360935	2010	Up	qRT-PCR
miR-210	PC	Stool	China	22504911	2012	Up	qRT-PCR
miR-210	CRC	Tissue	China	24632577	2014	Up	Real-time qPCR
miR-211	PC	Tissue	Italy	23155457	2012	Down	qRT-PCR
miR-212	GC	Tissue	China	21987613	2011	Up	Real-time qRT-PCR
miR-214	LC	Tissue	China	23962428	2013	Down	Real-time qPCR
miR-214	Pca	Tissue	USA	24167554	2013	Down	Real-time qPCR
miR-214	GC	Tissue	China	24614175	2014	Down	Real-time qPCR
miR-214	CRC	Tissue	China	24616020	2014	Down	qRT-PCR
miR-215	CRC	Tissue	USA	21752725	2011	Down	Real-time qPCR
miR-215	GC	Tissue	China	22205577	2012	Down	Real-Time PCR
miR-215	CRC	Tissue	China	23532818	2013	Down	Real-time qPCR
miR-215	GC	Tissue	China	23981575	2014	Up	Real-time qPCR
miR-217	PC	Tissue	USA	23933230	2013	Down	Real-time qRT-PCR
miR-218	GC	Plasma	China	22860003	2012	Down	qRT-PCR

(*Continued*)

ID	Cancer Name	Sample Type	Origin	PMID	Year	Expression Pattern	Validation Method
miR-218	CRC	Tissue	China	24294377	2013	Up	qRT-PCR
miR-22	CRC	Tissue	China	22492279	2012	Down	Real-time qRT-PCR
miR-22	GC	Tissue	China	23786758	2013	Down	qRT-PCR
miR-221	Pca	Tissue	Germany	19585579	2010	Down	qRT-PCR
miR-221	CRC	Plasma	China	20880178	2010	Up	qRT-PCR
miR-221	GC	Serum	China	22432036	2012	Up	Real-time qRT-PCR
miR-221	GC	Plasma	China	23307259	2013	Up	qRT-PCR
miR-221	PC	Tissue	Japan	23329235	2013	Up	qRT-PCR
miR-223	GC	Plasma	China	22860003	2012	Up	qRT-PCR
miR-223	NC	Serum	China	23056289	2012	Down	Real-time qPCR
miR-223-3p	LC	Plasma	Turkey	24595450	2014	Down	qRT-PCR
miR-224	Pca	Tissue	China	24382668	2013	Down	qRT-PCR
miR-224	Pca	Tissue	Greece	23136246	2013	Down	Real-time qPCR
miR-23a	CRC	Tissue	China	22455847	2012	Up	ISH
miR-25	CRC	Tissue	China	24293092	2014	Up	Real-time qPCR
miR-25	GC	Plasma	China	24595006	2014	Up	qRT-PCR
miR-25	EC	Tissue	China	24651474	2014	Up	qRT-PCR
miR-26a	LC	Tissue	China	23389848	2013	Down	Real-time qPCR
miR-26a	LC	Tissue	China	24259426	2013	Up	Real-time qPCR
miR-26a	TSCC	Tissue	China	24343426	2013	Down	qRT-PCR
miR-27a	GC	Serum	China	21112772	2011	Up	qRT-PCR
miR-27a	GC	Plasma	China	24122958	2014	Up	qRT-PCR
miR-27a-3p	PC	Peripheral blood mononuclear cells	China	23430754	2013	Up	qRT-PCR
miR-29	GC	Tissue	China	24130168	2014	Down	Real-time qPCR
miR-29a	CRC	Plasma	China	19876917	2010	Up	Real-time qRT-PCR
miR-29a	Leukaemia	Peripheral blood mononuclear cells	China	21678057	2012	Down	Real-time qRT-PCR
miR-29a	CRC	Serum	China	22018950	2012	Up	Real-time qRT-PCR
miR-29a	Leukaemia	Bone marrow mononuclear cells	China	22981932	2013	Down	Real-time qPCR

(*Continued*)

ID	Cancer Name	Sample Type	Origin	PMID	Year	Expression Pattern	Validation Method
miR-29a	CRC	Tissue	Spain	23673725	2013	Up	Real-time qPCR
miR-29a-5p	LC	Tissue	China	23285022	2012	Up	Real-time qPCR
miR-29b	Pca	Cell lines	USA	22402125	2012	Down	Real-time qRT-PCR
miR-29c	NC	Serum	China	23056289	2012	Down	Real-time qPCR
miR-30c	Pca	Tissue	China	24452717	2014	Down	Real-time qPCR
miR-30d	Pca	Cell lines	Japan	23231923	2012	Up	Real-time qRT-PCR
miR-31	EC	Tissue	China	21658006	2011	Up	Real-time qRT-PCR
miR-31	EC	Tissue	NA	22302717	2012	Down	Real-time qPCR
miR-31	GC	Tissue	China	19598010	2010	Down	Real-time qRT-PCR
miR-32	GC	Tissue	China	21874264	2011	Up	Real-time qRT-PCR
miR-32	Pca	Cell line Tissue	Finland	22266859	2012	Up	qRT–PCR
miR-32	CRC	Tissue	China	24123284	2013	Up	Real-time qRT-PCR
miR-320a	GC	Serum	China	23521833	2013	Down	qRT-PCR
miR-335	GC	Tissue	China	22802949	2012	Up	Real-time qRT-PCR
miR-335	GEDC	Tissue	China	24250228	2013	Down	Real-time qRT-PCR
miR-338-3p	GC	Cell lines	China	23826132	2013	Down	qPCR
miR-34	GC	Serum	China	21112772	2011	Up	qRT-PCR
miR-342	Leukaemia	Plasma	Belgium	23391324	2013	Down	real-time qRT-PCR
miR-34a	CRC	Tissue	China	23355243	2013	Down	RT-PCR
miR-34a	Pca	Cell lines	-	20687223	2010	Up	Real-time qRT-PCR
miR-362-3p	CRC	Tissue	Denmark	23280316	2013	Up	RT-qPCR
miR-363	CRC	Tissue	China	24519049	2014	Down	qRT-PCR
miR-372	LC	Tissue	China	23291979	2013	Up	qRT-PCR
miR-374b	Pca	Tissue	China	24191917	2013	Down	qRT-PCR
miR-375	EC	Tissue	USA and Canada	19789312	2009	Down	qRT-PCR
miR-375	GC	Tissue	China	21343377	2011	Up	Real-time qPCR
miR-375	EC	Tissue	NA	22302717	2012	Down	Real-time qPCR
miR-375	Pca	Serum	USA	22887127	2013	Up	Real-time qPCR
miR-375	GC	Tissue	China	23461060	2012	Down	real-time qRT-PCR
miR-375	Leukaemia	Bone marrow mononuclear cells	China	23864342	2013	Up	Real-time qRT-PCR
miR-375	PC	Tissue	China	24648956	2013	Down	Real-time PCR

(*Continued*)

ID	Cancer Name	Sample Type	Origin	PMID	Year	Expression Pattern	Validation Method
miR-376c	GC	Serum	China	22432036	2012	Up	Real-time qRT-PCR
miR-378	GC	Serum	China	22169097	2012	Up	Real-time qRT-PCR
miR-378	CRC	Tissue	Italy	24423916	2014	Up	qRT-PCR
miR-378*	Pca	Serum	USA	22887127	2013	Up	Real-time qPCR
miR-409-3p	Pca	Serum	USA	22887127	2013	Down	Real-time qPCR
miR-409-3p	Leukaemia	Bone marrow; Peripheral blood	Spain	24072101	2013	Down	Real-time qPCR
miR-421	GC	Tissue	China	19802518	2010	Up	RT-PCR
miR-421	GC	Plasma	China	22263628	2012	Up	Real-time qRT-PCR
miR-421	GC	Gastric juices	China	22926798	2012	Down	Real-time qRT-PCR
miR-423-5p	GC	Serum	China	21112772	2011	Up	qRT-PCR
miR-429	CRC	Tissue	China	23111103	2013	Up	Real-time qRT-PCR
miR-429	CRC	Tissue	China	24237355	2014	Down	Real-time qPCR
miR-429	CRC	Tissue	Spain	24510588	2014	Down	TaqMan Expression Assay
miR-449a	CRC	Tissue	China	24396489	2014	Up	Real-time qPCR
miR-451	GC	Tissue	Israel	22046085	2011	Up	Real-Time qPCR
miR-451	GC	Plasma	China	24595006	2014	Up	qRT-PCR
miR-4723	Pca	Tissue	USA	24223753	2013	Down	Real-time qPCR
miR-483-5p	LC	Plasma	USA	24127413	2013	Up	Real-time qPCR
miR-486-5p	GC	Plasma	China	24595006	2014	Up	qRT-PCR
miR-490-5p	CRC	Tissue	China	24519049	2014	Down	qRT-PCR
miR-492	LC	Tissue	Germany	21319197	2011	Up	Real-time qPCR
miR-548d	PC	Cell line	Germany	21946813	2012	Up	-
miR-574-3p	GC	Tissue	China	22683180	2012	Down	Real-time qPCR
miR-601	CRC	Plasma	China	22970209	2012	Down	qRT-PCR
miR-625	CRC	Tissue	China	23861214	2013	Down	qRT-PCR
miR-628-5p	Pca	Serum	USA	24477576	2014	Down	qRT-PCR
miR-630	GC	Tissue	China	24621930	2014	Up	Real-time qPCR
miR-638	GC	Plasma	China	24623314	2014	Down	Real-time qRT-PCR
miR-644	Leukaemia	Bone marrow; Peripheral blood	Spain	24072101	2013	Up	Real-time qPCR
miR-648	Pca	Tissue	China	24618011	2014	Down	qPCR
miR-708	Pca	Cell lines	USA	22552290	2012	Down	qRT-PCR

(*Continued*)

ID	Cancer Name	Sample Type	Origin	PMID	Year	Expression Pattern	Validation Method
miR-744	GC	Serum	China	22432036	2012	Up	Real-time qRT-PCR
miR-760	CRC	Plasma	China	22970209	2012	Down	qRT-PCR
miR-888	Pca	Cell lines	USA	24200968	2014	Up	qRT-PCR
miR-9	GEDC	Bile	Japan	21858175	2011	Up	High-throughput real-time PCR-based miRNA microarrays
miR-9	NC	Serum	China	24327016	2014	Down	qRT-PCR
miR-92	CRC	Tissue	China	19201770	2009	Up	Real-time qRT-PCR
miR-92a	CRC	Plasma	China	19876917	2010	Up	Real-time qRT-PCR
miR-92a	Leukaemia	Peripheral blood mononuclear cells	Japan	21182798	2010	Down	qRT-PCR
miR-92a	CRC	Stool samples	China	21930727	2013	Up	Real-time qRT-PCR
miR-92a	CRC	Tissue	China	22772712	2013	Up	Real-time qRT-PCR
miR-92a	CRC	Serum	China	23625654	2013	Up	real-time qRT-PCR
miR-92a	GC	Tissue	China	23868977	2013	Down	Real-time qPCR
miR-92a	GC	Plasma	China	24595006	2014	Up	qRT-PCR
miR-92a/ miR-638	Leukaemia	Whole blood	Japan	19440243	2009	Down	qRT-PCR
miR-93	GC	Tissue	China	22567743	2012	Up	Real-time qRT-PCR
miR-96	Pca	Tissue	China	20873592	2010	Down	ISH and TMA
miR-96	Leukaemia	Bone marrow or peripheral blood	China	24678958	2014	Down	Real-time qRT-PCR
miR-98	Pca	Tissue	China	20873592	2010	Down	ISH and TMA
miR-98	Pca	Cell lines	USA	23188821	2013	Up	qPCR

126 microRNA Biomarkers were collected from the literature that reported in 11 types of cancers.
ISH: in situ hybridization
TMA: technology of tissue microarray
qRT-PCR: quantitative reverse transcriptase-PCR
qPCR: quantitative PCR
TSCC: Tongue squamous cell carcinoma
PCa: Prostate cancer
CRC: Colorectum cancer
GC: Gastric Cancer
EC: Esophagus cancer
GEDC: Gallbladder and extrahepatic ducts cancer
LAC: Larynx cancer
LC: Liver cancer
PC: Pancreatic cancer

Supplementary Table S5. Edge list of Pediatric AML Specific microRNA-mRNA network [&]

microRNA	Gene
let-7a	APP
let-7a	LIN28A
let-7a	GNG5
let-7a	ACP1
let-7a	C14orf2
let-7a	RAVER2
let-7a	E2F1
let-7a	HNRPDL
let-7a	PDIA3
let-7a	CCT3
let-7a	SNRPD1
let-7a	KRAS
let-7a	RASAL2
let-7a	TMEM66
let-7a	ACTL6A
let-7a	RPL35A
let-7a	NARS2
let-7f	APP
let-7f	PDIA3
let-7f	NARS2
let-7f	FDPS
let-7f	ZBTB10
let-7f	TMEM66
let-7f	SNRPD1
let-7f	UFC1
let-7f	RASAL2
let-7f	GNG5
let-7f	ACTL6A
let-7f	CCT3
miR-10a	RABEP1
miR-10a	RAP2A
miR-10a	ATP5F1
miR-10a	GTF2I
miR-10a	SMC1A
miR-10a	ST7L

(*Continued*)

microRNA	Gene
miR-10a	WRN
miR-10a	SEC61B
miR-10a	MKL2
miR-10a	TRIM2
miR-10a	LYZL6
miR-10a	TRO
miR-10a	LANCL1
miR-10a	ASPH
miR-10a	PDCL
miR-10a	SMCR7L
miR-10a	TMEM62
miR-10a	MTMR6
miR-10a	NSL1
miR-10a	RAPGEF2
miR-10a	ELOVL6
miR-10a	HLA-DPB1
miR-10a	SNX4
miR-10a	TCP1
miR-10a	USP34
miR-10a	H3F3B
miR-10a	GRAMD1B
miR-10a	E2F3
miR-10a	TSEN34
miR-10a	USF2
miR-10a	RPS29
miR-10a	MTF2
miR-10a	SS18L2
miR-10a	IGBP1
miR-10a	ELAVL2
miR-155	PRKCI
miR-155	HNRNPA3P1
miR-155	HNRNPA3P1
miR-155	CUX1
miR-155	RCN2
miR-155	TWF1
miR-155	PAPOLA
miR-155	MLH1

(*Continued*)

microRNA	Gene
miR-155	RAPGEF2
miR-155	TRIP13
miR-155	FADS3
miR-155	TRIM32
miR-155	SH3BP4
miR-155	MSH6
miR-155	SMAD5
miR-155	KIAA0776
miR-155	POLE3
miR-155	KRAS
miR-155	SALL1
miR-155	PSMG1
miR-155	CAMTA1
miR-155	LDOC1
miR-155	CHAF1A
miR-155	FGF7
miR-155	VEZF1
miR-155	SMAD2
miR-155	DHX40
miR-155	MSH2
miR-155	DCAF7
miR-155	FADS1
miR-155	TBCA
miR-155	PKN2
miR-155	TMEM66
miR-155	UBE2J1
miR-155	MATR3
miR-155	ZNF236
miR-155	PELI1
miR-155	SMAD1
miR-155	DSG2
miR-16	GALNT7
miR-16	RECK
miR-16	KPNA3
miR-16	H3F3B
miR-16	TBP
miR-16	UBE2S

(*Continued*)

microRNA	Gene
miR-16	COPS2
miR-16	PPM1D
miR-16	TARBP2
miR-16	CCNT2
miR-16	PTCD3
miR-16	WBP5
miR-16	PNN
miR-16	NIPAL2
miR-16	CSHL1
miR-16	RAD51C
miR-16	SHOC2
miR-16	YTHDC1
miR-16	RARS
miR-16	PSMD7
miR-16	RBX1
miR-16	SLC7A1
miR-16	CEP63
miR-16	PLSCR4
miR-16	SLC12A2
miR-16	CEP55
miR-16	EIF4E
miR-16	MRPL20
miR-16	CDC23
miR-16	GPATCH8
miR-16	POLR3F
miR-16	DBNDD2
miR-16	PANX1
miR-16	PRIM1
miR-16	CRHBP
miR-16	AURKB
miR-16	NPR3
miR-16	HSPA4L
miR-16	RSBN1
miR-16	BRCA1
miR-16	MSH2
miR-16	TOMM34

(*Continued*)

microRNA	Gene
miR-16	UGDH
miR-16	CHORDC1
miR-16	HBXIP
miR-16	SIAH1
miR-16	CDC14B
miR-16	C4orf27
miR-16	PMS1
miR-16	C2orf43
miR-16	ARL2
miR-16	ITGA2
miR-16	SERPINE2
miR-16	ZBTB10
miR-16	WBP11
miR-16	ISOC1
miR-16	STXBP3
miR-16	HSDL2
miR-16	TMEM100
miR-181b	PDIA6
miR-181b	FBXO34
miR-181b	RNF6
miR-181b	PLAG1
miR-181b	C20orf29
miR-181b	SS18L2
miR-181b	TMF1
miR-181b	ZFAND6
miR-181b	CDCA4
miR-181b	PAWR
miR-181b	GRIA2
miR-181b	PRDM4
miR-191	GAP43
miR-191	REPS1
miR-191	SOX4
miR-191	SEH1L
miR-191	LUC7L2
miR-191	SP3
miR-191	TOMM20
miR-191	PEG10

(*Continued*)

microRNA	Gene
miR-191	CAND1
miR-191	SF1
miR-191	FUBP3
miR-191	BCL11A
miR-191	ATP5F1
miR-191	SLC23A2
miR-191	YAP1
miR-191	TSPAN13
miR-191	ELOVL5
miR-191	SMARCA4
miR-191	MORF4L2
miR-191	HNRPDL
miR-191	DUSP11
miR-191	CAPZA1
miR-191	TIMM17A
miR-191	PDE6D
miR-191	USP34
miR-191	MORF4L1
miR-191	RNF139
miR-196b	PER1
miR-196b	SMC3
miR-196b	SSR2
miR-196b	WASF1
miR-196b	RPL39L
miR-196b	SLC35D2
miR-196b	RAPGEF5
miR-196b	TSPAN12
miR-196b	ZZZ3
miR-196b	TSPAN3
miR-196b	KHDRBS3
miR-196b	CRIM1
miR-196b	CYP4B1
miR-196b	PRMT3
miR-196b	SMC1A
miR-196b	ANKHD1
miR-196b	PDE6D
miR-196b	TMEM135

(*Continued*)

microRNA	Gene
miR-196b	LANCL1
miR-196b	DYNC2LI1
miR-196b	HEPH
miR-196b	TMEM143
miR-196b	RBM12
miR-196b	NME4
miR-196b	DYRK3
miR-196b	BCL11A
miR-196b	KCNJ2
miR-196b	IGFBP7
miR-196b	BCL7C
miR-196b	SNX16
miR-196b	NUP54
miR-196b	WNT5A
miR-196b	SSR1
miR-196b	ZNF281
miR-196b	RBM26
miR-196b	ARID5B
miR-196b	TRPC3
miR-196b	TMEM66
miR-196b	E2F3
miR-196b	ASPH
miR-196b	SRP9
miR-196b	SMCR7L
miR-196b	RAD23B
miR-196b	EPHA7
miR-196b	SNX4
miR-196b	RNF138
miR-196b	TMEM135
miR-196b	LDOC1
miR-19a	ING3
miR-19a	NME4
miR-19a	MED4
miR-19a	PRKAA1
miR-19a	ZC3H15
miR-19a	MREG
miR-19a	LSM3

(*Continued*)

microRNA	Gene
miR-19a	PRMT5
miR-19a	SMAD4
miR-19a	CAMTA1
miR-19a	FOXF2
miR-19a	RAPGEF2
miR-19b	RAPGEF2
miR-19b	PRKAA1
miR-19b	ARID4B
miR-19b	CAMTA1
miR-19b	VPS37B
miR-19b	FOXF2
miR-19b	SMARCD2
miR-19b	NME4
miR-19b	LSM3
miR-19b	MREG
miR-19b	RBBP7
miR-19b	SOX4
miR-19b	CLIP1
miR-19b	SLMO2
miR-20a	CROT
miR-20a	ATAD2
miR-20a	APP
miR-20a	BTG3
miR-20a	MICA
miR-20a	HIF1A
miR-20a	C19orf2
miR-20a	DDX5
miR-20a	E2F1
miR-20a	SGCE
miR-20a	E2F5
miR-20a	BNIP2
miR-20a	SMAD4
miR-20a	CENPQ
miR-20a	MRPL24
miR-20a	WBSCR22
miR-20a	PTPN4
miR-20a	SRP9

(*Continued*)

microRNA	Gene
miR-20a	RNH1
miR-20a	GLO1
miR-20a	SACS
miR-20a	KIF23
miR-218	ITM2C
miR-218	MRPS27
miR-218	ARID4B
miR-218	TPD52
miR-218	RNF38
miR-218	RPL35
miR-218	EBP
miR-218	SSR1
miR-221	EIF1
miR-221	GPR107
miR-221	TCEAL1
miR-221	SSRP1
miR-221	DDIT4
miR-223	CENPN
miR-223	E2F1
miR-223	RBM3
miR-223	STMN1
miR-223	CCT3
miR-223	NDUFA5
miR-223	ZNF330
miR-223	RCN2
miR-25	GRAMD1B
miR-25	FHL2
miR-25	NFIB
miR-25	VBP1
miR-25	CNNM1
miR-25	NOX4
miR-25	ANP32E
miR-25	NOVA1
miR-25	E2F3
miR-25	ITPR1
miR-25	TEAD1

(*Continued*)

microRNA	Gene
miR-25	QSER1
miR-25	GALNT7
miR-25	FBXW7
miR-25	ADCY3
miR-25	DDIT4
miR-25	CIC
miR-25	TRIM36
miR-25	KLF12
miR-25	H3F3B
miR-25	PRDX3
miR-25	SERP1
miR-25	CCNC
miR-25	GPATCH8
miR-25	ZNF281
miR-25	NEFL
miR-25	CCNT2
miR-25	SLC7A11
miR-25	VDAC2
miR-25	PRPF40A
miR-25	SOX4
miR-25	BUB3
miR-25	RSBN1
miR-25	BCL11A
miR-25	KIAA1045
miR-25	FMR1
miR-25	DYRK2
miR-25	ATXN3
miR-25	ZNF238
miR-25	PA2G4
miR-25	MOAP1
miR-25	PRMT5
miR-25	PCDH11X
miR-25	MYCBP2
miR-25	GAP43
miR-25	COPS2
miR-25	TMF1
miR-25	SIP1

(*Continued*)

microRNA	Gene
miR-25	STAG2
miR-25	CAMTA1
miR-25	SLC25A32
miR-25	CAND1
miR-25	HBS1L
miR-25	FAM120A
miR-25	CCT6A
miR-25	SMAD7
miR-25	HIVEP1
miR-25	PBLD
miR-25	ZNF804A
miR-25	DNAJB9
miR-25	ING3
miR-25	SLC6A1
miR-26b	DERL2
miR-26b	DDX52
miR-26b	MTX2
miR-26b	EPB41L3
miR-26b	KCNK1
miR-26b	NFYB
miR-26b	SFPQ
miR-26b	UBE2B
miR-26b	CNBP
miR-26b	METAP2
miR-26b	SLC19A2
miR-26b	LPHN2
miR-26b	TXNL1
miR-26b	RBBP6
miR-26b	LAP3
miR-26b	ZBTB10
miR-26b	SERP1
miR-26b	DDAH1
miR-26b	ART3
miR-26b	PAWR
miR-26b	TTK
miR-26b	B4GALT4
miR-26b	EZH2

(*Continued*)

microRNA	Gene
miR-26b	MTMR4
miR-26b	SLC7A11
miR-26b	RCN2
miR-26b	TJP2
miR-26b	DHX29
miR-26b	NEFL
miR-26b	RAB28
miR-26b	PDHX
miR-26b	CREBZF
miR-26b	SFPQ
miR-26b	SNX7
miR-26b	WNT5A
miR-26b	PLOD2
miR-26b	NUP153
miR-26b	NUP54
miR-26b	CAPZA1
miR-26b	KIAA0947
miR-26b	SMAP1
miR-26b	OBFC2A
miR-26b	COX5A
miR-26b	PSMD10
miR-26b	FAM98A
miR-26b	CLTC
miR-26b	WBP11
miR-26b	RNF6
miR-26b	LEF1
miR-26b	COPS2
miR-26b	PTS
miR-26b	15-Sep
miR-26b	ING3
miR-26b	PDE4B
miR-26b	MDH1
miR-26b	BCL7B
miR-26b	KIF23
miR-26b	OAT
miR-26b	MTMR1
miR-26b	ME1

(*Continued*)

microRNA	Gene
miR-26b	RUFY3
miR-26b	COPS6
miR-26b	UCHL5
miR-26b	ATP5C1
miR-26b	PNRC1
miR-26b	ZNF259
miR-26b	ELAVL2
miR-26b	IARS
miR-26b	AGPAT5
miR-26b	SMC1A
miR-26b	RNMT
miR-26b	CHORDC1
miR-26b	SLBP
miR-26b	ZNF238
miR-26b	KPNA3
miR-26b	CKS2
miR-26b	SCFD1
miR-26b	SACS
miR-26b	TMEM66
miR-26b	BCL11A
miR-26b	ATP6V1G1
miR-425	RAPGEF2
miR-425	BTG3
miR-425	BACH2
miR-425	TRO
miR-425	STMN1
miR-425	MAP3K4
miR-425	ZFAND1
miR-425	PSIP1
miR-425	TERF1
miR-425	B4GALT4
miR-425	SMAD2
miR-425	PTGER3
miR-425	WTAP
miR-425	NSL1
miR-425	RINT1
miR-425	MDM1

(*Continued*)

microRNA	Gene
miR-425	QSER1
miR-425	ATP5G3
miR-425	ASPH
miR-425	EBNA1BP2
miR-425	SLC16A1
miR-425	RABAC1
miR-425	BCOR
miR-425	FAM63B
miR-425	ELOVL5
miR-425	CCNT2
miR-425	PTP4A2
miR-425	CREBZF
miR-425	PRDX6
miR-425	MAP9
miR-425	PGAP1
miR-425	NOTCH1
miR-92a	SMAD4
miR-92a	MYCBP2
miR-92a	HBS1L
miR-92a	CIC
miR-92a	ANP32E
miR-92a	PUS7
miR-92a	TMF1
miR-92a	ARID4B
miR-92a	SIP1
miR-92a	FHL2
miR-92a	ITPR1
miR-95	RINT1
miR-95	PPP1CC
miR-95	YEATS4
miR-95	PSMD10
miR-95	VEZF1
miR-95	SOX4
miR-95	SF1
miR-95	USP6NL
miR-95	LEMD3

(*Continued*)

microRNA	Gene
miR-95	SMAD4
miR-95	EID1
miR-95	FHL2
miR-95	SERP1
miR-95	TRPM3
miR-95	KIAA0528
miR-95	YTHDF2
miR-95	FASTKD2
miR-95	RBBP7
miR-95	CYP39A1
miR-95	EGLN3
miR-95	SLK
miR-95	PDE4B
miR-95	KCND2
miR-95	DNAJC15
miR-95	QKI
miR-95	PIGA
miR-95	ARID4B

[&]The genes listed here is the unique target of the microRNA, here the unique target means the gene is only targeted by the microRNA and not by other microRNAs in the AML microRNA-mRNA network. The network was constructed by integrating the microRNA-mRNA network, microRNA expression data and mRNA expression data. Therefore the unique target sets is related to the concrete network in context.

Supplementary Table S6. Candidate microRNA biomarkers and their target genes

microRNA-196b Targets			microRNA-155 Targets			microRNA-25 Targets		
Gene	Unique Target	TF	Gene	Unique Target	TF	Gene	Unique Target	TF
ANKHD1	T	F	CAMTA1	F	T	ADCY3	T	F
ARID5B	T	T	CHAF1A	T	F	ANP32E	F	F
ASPH	F	F	CUX1	T	T	ATXN3	T	F
BCL11A	F	T	DCAF7	T	F	BCL11A	F	T
BCL7C	T	F	DHX40	T	F	BUB3	T	F
CRIM1	T	F	DSG2	T	F	CAMTA1	F	T
CYP4B1	T	F	FADS1	T	F	CAND1	F	F
DYNC2LI1	T	F	FADS3	T	F	CCNC	T	F
DYRK3	T	F	FGF7	T	F	CCNT2	F	F
E2F3	F	T	HNRNPA3P1	F	F	CCT6A	T	F
EPHA7	T	F	KIAA0776	T	F	CIC	F	T
HEPH	T	F	KRAS	F	F	CNNM1	T	F
IGFBP7	T	F	LDOC1	F	F	COPS2	F	T
KCNJ2	T	F	MATR3	T	T	DDIT4	F	F
KHDRBS3	T	F	MLH1	T	F	DNAJB9	T	F
LANCL1	F	F	MSH2	F	F	DYRK2	T	F
LDOC1	F	F	MSH6	T	F	E2F3	F	T
NME4	F	F	PAPOLA	T	F	FAM120A	T	F
NUP54	F	F	PELI1	T	F	FBXW7	T	F
PDE6D	F	F	PKN2	T	F	FHL2	F	F
PER1	T	F	POLE3	T	T	FMR1	T	F
PRMT3	T	T	PRKCI	T	F	GALNT7	F	F
RAD23B	T	F	PSMG1	T	F	GAP43	F	F
RAPGEF5	T	T	RAPGEF2	F	F	GPATCH8	F	T
RBM12	T	F	RCN2	F	F	GRAMD1B	F	F
RBM26	T	T	SALL1	T	T	H3F3B	F	F
RNF138	T	T	SH3BP4	T	F	HBS1L	F	F
RPL39L	T	F	SMAD1	T	T	HIVEP1	T	T
SLC35D2	T	F	SMAD2	F	T	ING3	F	F
SMC1A	F	F	SMAD5	T	T	ITPR1	F	F
SMC3	T	F	TBCA	T	F	KIAA1045	T	F
SMCR7L	F	F	TMEM66	F	F	KLF12	T	T
SNX16	T	F	TRIM32	T	T	MOAP1	T	F
SNX4	F	F	TRIP13	T	F	MYCBP2	F	F

microRNA-196b Targets			microRNA-155 Targets			microRNA-25 Targets		
Gene	Unique Target	TF	Gene	Unique Target	TF	Gene	Unique Target	TF
SRP9	F	F	TWF1	T	F	NEFL	F	F
SSR1	F	F	UBE2J1	T	F	NFIB	T	T
SSR2	T	F	VEZF1	F	T	NOVA1	T	F
TMEM135	F	F	ZNF236	T	T	NOX4	T	F
TMEM143	T	F				PA2G4	T	F
TMEM66	F	F				PBLD	T	F
TRPC3	T	F				PCDH11X	T	F
TSPAN12	T	F				PRDX3	T	F
TSPAN3	T	F				PRMT5	F	F
WASF1	T	F				PRPF40A	T	F
WNT5A	F	F				QSER1	F	F
ZNF281	F	T				RSBN1	F	F
ZZZ3	T	T				SERP1	F	F
						SIP1	F	F
						SLC25A32	T	F
						SLC6A1	T	F
						SLC7A11	F	F
						SMAD7	T	T
						SOX4	F	T
						STAG2	T	F
						TEAD1	T	T
						TMF1	F	F
						TRIM36	T	F
						VBP1	T	F
						VDAC2	T	F
						ZNF238	F	T
						ZNF281	F	T
						ZNF804A	T	T

T: True; F: False

Supplementary Table S7. qRT-PCR results for microRNA-196b expression in different AML samples and the control samples

M4~5		non-M4~5		Not determined		Control
Diagnosis	$2^{-\Delta\Delta Ct}$	Diagnosis	$2^{-\Delta\Delta Ct}$	Diagnosis	$2^{-\Delta\Delta Ct}$	$2^{-\Delta\Delta Ct}$
M5	13.73355607	M6	8.798758634	AML	0.025766374	1.023042914
M4	6.531736825	M2	25.06843446	AML	5.420903519	3.933553839
M4	0.007044274	M2	10.45605452	AML	8.222250523	0.193897575
M5	4.40874081	M2	0.701314436			1.744483554
M5	4.758367383	M3	0.612154051			1.391621297
M4	0.057088739	M2	0.09154765			2.946631525
M5	14.60087911	M2	0.036207106			0.641846402
M5	11.15846492	M2	0.037499539			0.953386651
M5	15.76441792	M2	3.469353057			1.5017729
M4	0.183133378	M2	0.026233425			2.578859777
M5	1.309001421	M2	0.005072221			2.408052195
M5	14.61623669	M2	0.009382144			1.778548106
M4	12.67839307	M2	0.042303866			0.111349475
M4	13.17562959	M2	0.012204018			0.977476098
M5	0.017354056	M2	0.005311555			0.7792726
M5	67.41199098					2.286223842
						1.061913802
						0.955404336
						0.776074288
						1.550678921
						0.812515175
						1.35250444
						0.928549031
						0.494074327
						0.842140778
						1.064985162
						0.292015461
						1.143745973
						0.29308682
						0.492615296

Supplementary Table S8. Previous Report about Candidates microRNAs in AML

MicroRNA	Location	Family	Expression	Sample	Experiment vs. Control
miRNA-196b	7p15	196	Up regulated	Adults	AML vs. ALL (1)
					FLT3 AML vs. other AML (2)
					NPM1 AML vs. other AML (2, 3)
					MLL rearrangements AML vs. other AML (4)
				Children	FLT3, NPM1, or MLL rearrangements AML vs. other AML (5)
					MLL rearrangements AML vs. other AML (6)
miRNA-155	21q21	155	Up regulated	Adults	AML vs. healthy donors (bone marrow) (7, 8)
					FLT3 AML vs. other AML(3, 9, 10)
					NPM1 AML vs. other AML (2)
				Children	FLT3 or NPM1 AML vs. other AML (5)

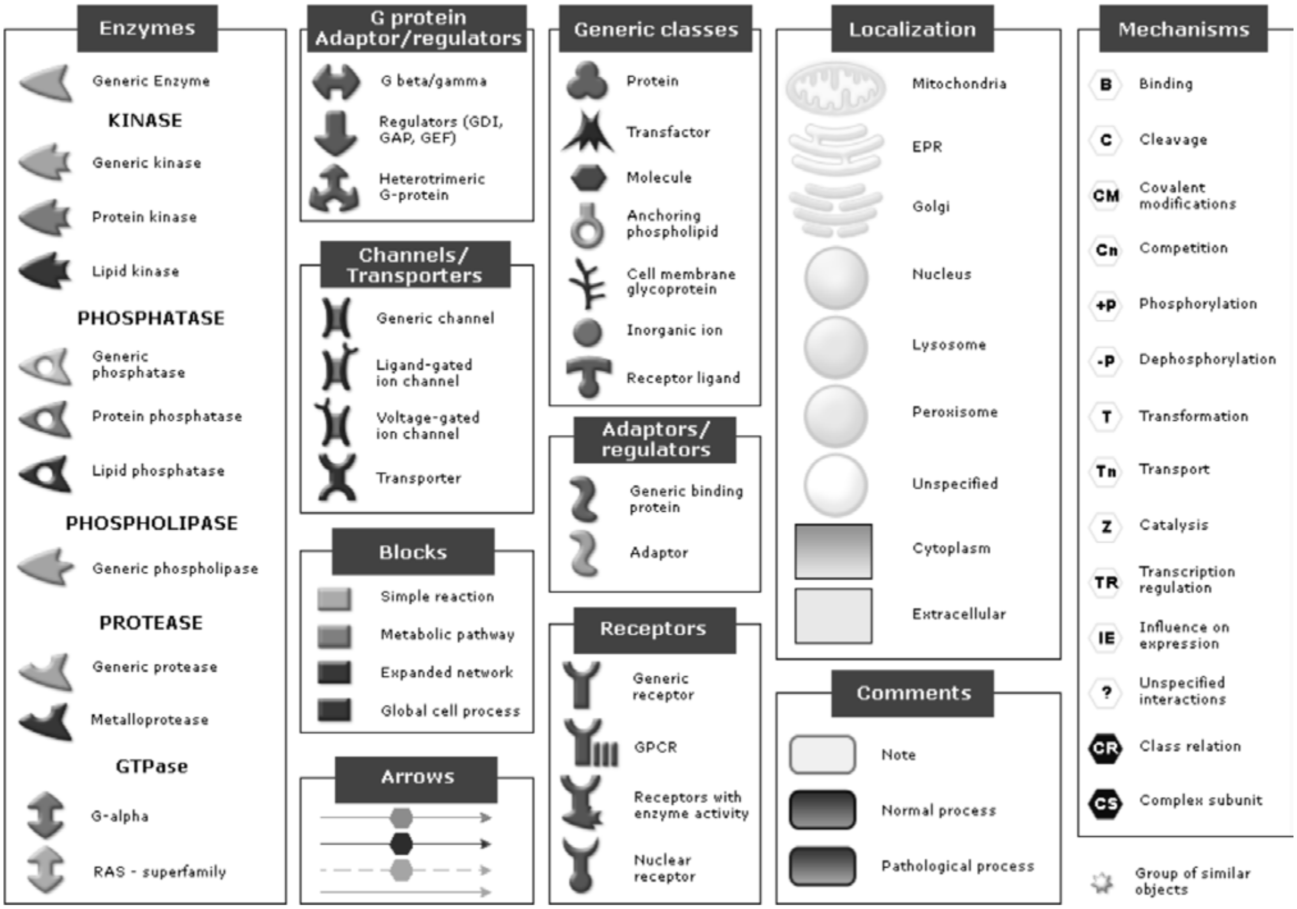

Supplementary Figure S1: Detailed figure legend for MetaCore (GeneGo) pathway maps and networks http://www.genego.com/

REFERENCES

1. Wang, Y., Z. Li, C. He, D. Wang, X. Yuan, J. Chen, et al. MicroRNAs expression signatures are associated with lineage and survival in acute leukemias. Blood Cells Mol Dis. 2010; 44:191–7.
2. Cammarata, G., L. Augugliaro, D. Salemi, C. Agueli, M. La Rosa, L. Dagnino, et al. Differential expression of specific microRNA and their targets in acute myeloid leukemia. Am J Hematol. 2010; 85:331–9.
3. Jongen-Lavrencic, M., S.M. Sun, M.K. Dijkstra, P.J. Valk, B. Lowenberg. MicroRNA expression profiling in relation to the genetic heterogeneity of acute myeloid leukemia. Blood. 2008; 111:5078–85.
4. Li, Z., J. Lu, M. Sun, S. Mi, H. Zhang, R.T. Luo, et al.Distinct microRNA expression profiles in acute myeloid leukemia with common translocations. Proc Natl Acad Sci U S A. 2008; 105:15535–40.
5. Danen-van Oorschot, A.A., J.E. Kuipers, S. Arentsen-Peters, D. Schotte, V. de Haas, J. Trka, et al. Differentially expressed miRNAs in cytogenetic and molecular subtypes of pediatric acute myeloid leukemia. Pediatr Blood Cancer. 2012; 58:715–21.
6. Daschkey, S., S. Rottgers, A. Giri, J. Bradtke, A. Teigler-Schlegel, G. Meister, et al. MicroRNAs distinguish cytogenetic subgroups in pediatric AML and contribute to complex regulatory networks in AML-relevant pathways. PLoS One. 2013; 8:e56334.
7. Dixon-McIver, A., P. East, C.A. Mein, J.B. Cazier, G. Molloy, T. Chaplin, et al. Distinctive patterns of microRNA expression associated with karyotype in acute myeloid leukaemia. PLoS One. 2008; 3:e2141.
8. O'Connell, R.M., D.S. Rao, A.A. Chaudhuri, M.P. Boldin, K.D. Taganov, J. Nicoll, et al.. Sustained expression of microRNA-155 in hematopoietic stem cells causes a myeloproliferative disorder. J Exp Med. 2008; 205:585–94.
9. Garzon, R., S. Volinia, C.G. Liu, C. Fernandez-Cymering, T. Palumbo, F. Pichiorri, et al. MicroRNA signatures associated with cytogenetics and prognosis in acute myeloid leukemia. Blood. 2008; 111:3183–9.
10. Garzon, R., M. Garofalo, M.P. Martelli, R. Briesewitz, L. Wang, C. Fernandez-Cymering, et al. Distinctive
